# From tissue engineering to mosquitoes: biopolymers as tools for developing a novel biomimetic approach to pest management/vector control

**DOI:** 10.1186/s13071-022-05193-y

**Published:** 2022-03-05

**Authors:** Marco Friuli, Claudia Cafarchia, Riccardo Paolo Lia, Domenico Otranto, Marco Pombi, Christian Demitri

**Affiliations:** 1 Department of Engineering for Innovation, University of Salento, 73100 Lecce, Italy; 2 Department of Veterinary Medicine, University of Bari, Valenzano, Italy; 3 Dipartimento Di Sanità Pubblica E Malattie Infettive, Università Di Roma “Sapienza”, Rome, Italy

**Keywords:** Integrated pest management, Vector control, Ovitrap, Biomaterial, Insecticide, Hydrogel, *Aedes albopictus*

## Abstract

**Background:**

Pest management has been facing the spread of invasive species, insecticide resistance phenomena, and concern for the impact of chemical pesticides on human health and the environment. It has tried to deal with them by developing technically efficient and economically sustainable solutions to complement/replace/improve traditional control methods. The renewal has been mainly directed towards less toxic pesticides or enhancing the precision of their delivery to reduce the volume employed and side effects through lure-and-kill approaches based on semiochemicals attractants. However, one of the main pest management problems is that efficacy depends on the effectiveness of the attractant system, limiting its successful employment to semiochemical stimuli-responsive insects. Biomaterial-based and bioinspired/biomimetic solutions that already guide other disciplines (e.g., medical sciences) in developing precision approaches could be a helpful tool to create attractive new strategies to liberate precision pest management from the need for semiochemical stimuli, simplify their integration with bioinsecticides, and foster the use of still underemployed solutions.

**Approach proposed:**

We propose an innovative approach, called “biomimetic lure-and-kill”. It exploits biomimetic principles and biocompatible/biodegradable biopolymers (e.g., natural hydrogels) to develop new substrates that selectively attract insects by reproducing specific natural environmental conditions (biomimetic lure) and kill them by hosting and delivering a natural biopesticide or through mechanical action. Biomimetic lure-and-kill-designed substrates point to provide a new attractive system to develop/improve and make more cost-competitive new and conventional devices (e.g. traps). A first example application is proposed using the tiger mosquito *Aedes albopictus* as a model.

**Conclusions:**

Biomaterials, particularly in the hydrogel form, can be a useful tool for developing the biomimetic lure-and-kill approach because they can satisfy multiple needs simultaneously (e.g., biomimetic lure, mechanical lethality, biocompatibility, and bioinsecticide growth). Such an approach might be cost-competitive, and with the potential for applicability to several pest species. Moreover, it is already technically feasible, since all the technologies necessary to design and configure materials with specific characteristics are already available on the market.

**Graphical Abstract:**

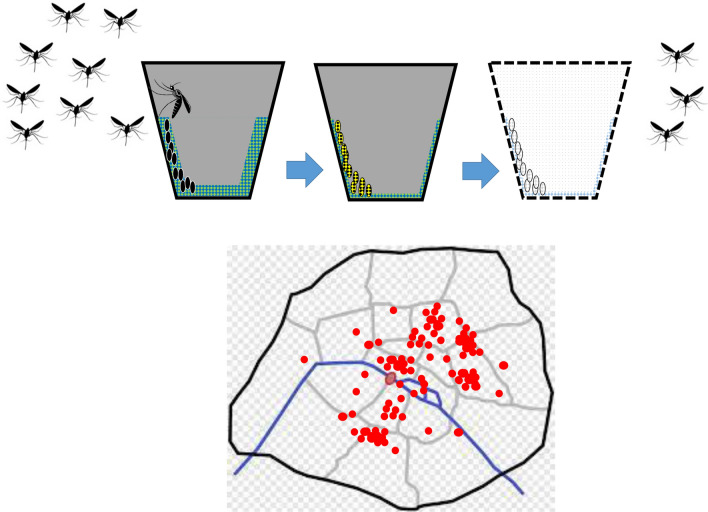

## Background

Biomaterials (BM), and in particular biopolymers (BP), have already played a crucial role in the birth and development of tissue engineering (TE), one of the most revolutionary approaches in regenerative medicine of the last 30 years. TE is based on imitation [1]. It combines and applies the principles of material engineering, life sciences, and biomimetics, and the properties of BMs (e.g., biocompatibility and biodegradation) to produce scaffolds that replicate the most critical and suitable physiological conditions to promote healing by specific cell proliferation/tissue restoration [1]. Then, scaffolds can be employed directly in the body to sustain regeneration or after the production (through cell seeding) and implantation of lab-grown new living and functional tissues [2].

TE relies on the finding that it is possible to manage and improve the outcome of a biological target (e.g., cell seeding) by choosing and manipulating the scaffold composition and features, based on the evidence that cell survival and production of physiologically functioning structures are related to tissue properties (chemical and physical). Thus, a scaffolding system has to be biomimetic, i.e. able to replicate physiological tissue behaviour, to allow seeded cells to survive and proliferate to heal damages or produce new tissue. Consequently, the design and production of biomimetic scaffolds are crucial for the success of TE [1]. BMs and BPs have been studied extensively and found to be more suitable for biomimetic scaffold preparation. The continuous improvement of innovative biocompatible materials processing and sources (e.g., polysaccharides and polypeptides) allowed the production of complex 3D biomimetic structures and scaffolds increasingly able to replicate the physiological mechanisms of transport and signalling [3] of human tissues. Furthermore, the constant improvement in knowledge of BPs has promoted advances in TE, such as in other medical therapies (e.g., drug delivery or targeting), and has led to precision and personalised medicine such as cancer therapy [4]. Consequently, there is extensive expertise to produce biomimetic and BM-based substrates that can be exploited in other fields.

In recent decades, pest management, particularly the control of disease vectors, has been faced with numerous new challenges, demanding technically and economically sustainable solutions to complement or replace traditional approaches. Among the significant issues, it is possible to account for the appearance and spread of invasive species in new geographical areas and habitats (mainly due to climate change and globalised movement of people and goods [5, 6]) and the emergence of insecticide resistance phenomena in pests and the consequent progressive loss of efficacy of principal molecules and control strategies [7, 8]. In addition, the impact of several insecticides on health and biodiversity, and generally a higher environmental sensibility, has led to increasingly restrictive regulations about legal molecules, pest management practices, and greater attention to devices' ecological impact [9, 10]. Some effective compounds are considered too risky to be used in settings associated with humans, animals, or foods (e.g. in the livestock or food industry). Consequently, the reduction in control solutions strategies pushes manufacturers, public health institutions, and researchers to develop innovative sustainable solutions to reduce insecticide volumes employed or involve biopesticides and novel pesticide-free control approaches.

Thus, pest management has slowly moved from a predominantly broad use of chemical insecticides to a more sophisticated path of “precision” approaches to improve targeting towards a specific insect group, with solutions that allow for an increasingly high environmental and health safety profile by reducing (and eliminating, when possible) quantities and toxicity of insecticides involved in the application. For example, a strategy such as insecticide spraying, the former gold standard of pest management, has ultimately been retained as a non-selective technique (and in some cases not very effective given the lack of targeting and delivery), and therefore its use is limited to strictly necessary applications (e.g., in vector-borne disease transmission areas [10–13]).

The lure-and-kill approach was among the first and more successful examples of precision control strategies [11]. It was designed to increase targeting and insecticide delivery mainly through insect attraction triggered by semiochemicals [12, 13] such as sex or food pheromones, odours, or natural/synthetic baits. Semiochemicals help convey the insecticidal agent more directly and effectively by attracting a specific insect on traps or toxic surfaces, or encouraging the ingestion of the toxic bait, as effectively shown for malaria mosquitoes [14, 15]. Although still based on the use of chemical insecticides, the precision approach represents a fundamental step forward to reduce the volumes of insecticides employed and facilitate their use indoors in the presence of people and/or animals or industrial disinfestation (e.g. food industry). However, this approach fails when species-specific attractants or food preferences are unavailable for the target pest. In these situations, the semiochemical approach can be ineffective because the insect ignores the attractant or it may generate repellent reactions [16]. Therefore, it is necessary to explore new precision control methods that do not exclusively use a semiochemical or feeding signal to trigger a behavioural reaction in the insect.

## Biomimetic lure-and-kill approach: how the knowledge in biomaterials can be useful in pest management

BMs, BPs, and TE expertise can help in developing new lure-and-kill strategies. For example, taking inspiration from TE, in particular from how artificial substrate features influence cell behaviour, it is possible to hypothesise new attractive systems that could exploit pests' repetitive behavioural patterns related to essential life-cycle activities such as reproductive habits or search for suitable oviposition sites. A possible approach could be based on stand-alone substrates or devices with the ability to replicate or mimic natural habitat features (physicochemical, morphological, mechanical) identified as involved in triggering a specific behaviour and thus influencing the attraction of the target pest species. At the same time, moving from the typical scaffolds' biocompatibility required to host cells, it is possible to suppose the integration of a natural living bioinsecticide (e.g., bacteria, fungi) inside the biomimetic substrates or to design it to perform a lethal mechanical action such as a sticky or viscous trapping material.

This approach can be called “biomimetic lure-and-kill”. Its critical elements are the identification of the key environmental features and the realisation of an artificial environment that mimics them as closely as possible (biomimetic artificial environment), which is at the same time compatible with the conditions for the survival and growth of the lethal agent that can perform the mechanical action.

Based on TE knowledge, this approach needs to rely on BPs in particular, which are more suitable for finely adjusting material properties to create multifunctional substrates. Moreover, BP biocompatibility can be exploited to host and promote the survival and metabolic activity of natural killing agents (i.e., bioinsecticides). Among them, polysaccharides such as cellulose and alginates are low-cost and naturally abundant BPs. They are also biodegradable, biocompatible, and processable to form super-absorbent hydrogels [17]. This particular semi-solid polymer state absorbs and slowly releases large amounts of water. This could be an essential feature to attract some classes of pests and, overall, allow the survival of those bioinsecticides that are exceptionally sensitive to drought and extreme environmental conditions, and then increase the operational time of the insecticidal device in field conditions. In fact, as already reported in regenerative medicine applications [18], if the polymers are appropriately configured, the hydrogel-induced microenvironment can host and promote cell growth and, therefore, the growth of insect-pathogenic microorganisms such as bacteria and fungi [19]. But, above all, hydrogels allow for easy adjustment of some of their parameters within a wide range of values, guaranteeing the possibility of imitating several different natural environments by minimally varying the composition or processing of the same polymer (e.g., by varying polymer type or other reagent concentration). The numerous fields of application confirm hydrogel versatility. However, outside of TE, hydrogels are mainly derived from oil-based polymers (e.g., polyacrylamide, PAM) and employed as controlled-release systems or for liquid absorption. For example, synthetic hydrogels are applied in agriculture for nutrient or pesticide release, for absorption and sustained release of water, particularly in dryland farming in soil-less agriculture, or hygiene products such as diapers [20, 21]. Synthetic hydrogels are already used in pest management, mainly for the release of chemical insecticides through beads as baits or for controlled release in water or microencapsulation for spraying and release of active substances [22, 23]. Examples are PAM or silica and talc-based gels [24, 25] inserted in a polyethylene container to slowly release an insect growth regulator or other synthetic insecticides in pest and vector control [26, 27]. Hydrogels, in some cases, have also been employed to improve biopesticide spraying [28, 29].

Although presenting all the advantages of precision pest management in terms of pesticide volume use, hydrogels as controlled insecticide release systems cannot be defined as a completely eco-compatible solution. For example, when employed in semiochemical-based lure-and-kill trapping devices, they do not solve the issue of pesticide release or the dispersion of synthetic polymers in the environment, thus failing to meet the green spirit required in developing new control methods [30]. Furthermore, the biomimetic lure-and-kill approach is based on a different problem–solution approach, in which the hydrogel is an attractive system and a lethal substrate at the same time and not employed only for controlled release and pesticide encapsulation. Adopting a BP-based technology would define a win–win solution for both manufacturers/stakeholders and the environment. An economic advantage could derive from greater efficacy of the product and, in the case of mechanical action, from the lack of a biocide, offering advantages in terms of registration and regulatory compliance. In addition, fully biodegradable devices could be potentially adopted as a control method in areas where traditional pesticide-based approaches are no longer applicable.

## Biomimetic approach as a possible solution to the limits of current *Aedes albopictus* control strategies

The control of the tiger mosquito, *Aedes albopictus* (Diptera: Culicidae), is an appropriate context in which the biomimetic approach might be helpful and successfully applied. *Aedes albopictus* is one of the major invasive species in the world [31], a vector of several arboviruses including dengue, chikungunya, and Zika [32, 33], imposing a heavy public health and economic burden, particularly in temperate areas [34–36]. It is a day-biting species primarily associated with anthropised contexts, typically resting in shady places during inactivity [37–39]. The tiger mosquito is a container-breeding mosquito that normally does not lay its eggs in water but rather on humid substrates in sites that will be subsequently flooded. In its original rural habitats, *A. albopictus* lays eggs in several substrates (e.g., trunks or walls of cavities present in plants or rocks) [40]. In contrast, in anthropised environments of temperate countries, the tiger mosquito is adapted to oviposition mostly on artificial habitats (cartons, trash containers, used tires, etc.) [41] in addition to natural breeding habitats such as green areas typically abundant in peridomestic settings [42, 43]. At the moment, numerous methods for controlling *A. albopictus* are adopted [44], spanning from chemical to biological and mechanical ones. Chemical methods, despite their pollution problems, lack of selectivity, and increasing insecticide resistance [45], are still among the most widely employed control methods. They involve mainly pyrethroids and insect growth regulators, respectively employed as adulticides and larvicides, which are space-sprayed or directly applied on potential breeding sites. Pyrethroids and insect growth regulators are unique chemicals used in Europe in mosquito control strategies, in accordance with Directive 98/8/EC (Biocidal Products Directive) and EU Regulation 528/2012 (Biocidal Products Regulation [BPR]). The direct application of adulticides can be very effective in favourable conditions, particularly against *A. albopictus* (and also *Aedes aegypti*). However, the efficacy of adulticidal direct application can be negatively affected by environmental conditions and the repellent effect of some insecticidal compounds [46–48], as well as by difficulties in spraying insecticides in private areas. Consequently, chemical methods, and in particular direct application of adulticidal substances, are effective when locally applied but not for wide areas, mainly because of their environmental impact [49–51]. In addition, due to the strong limits imposed by the BPR, adulticidal applications will probably no longer be permitted and will have to be replaced with other strategies. Biological approaches have been proposed as an alternative or complement to chemical methods. Bioinsecticides such as bacteria (e.g., *Bacillus thuringiensis* [*Bti*]), symbionts (e.g., *Wolbachia pipientis*), entomopathogenic fungi (e.g., *Beauveria bassiana* [*Bb*] and *Metarhizium anisopliae*) [52–55], and natural essential oils (e.g., oil of pennyroyal [*Mentha pulegium*] or *Ruta chalepensis*) [56–58] have been among the most commonly promoted. However, they may still present difficulties in targeting and selectivity when sprayed. Furthermore, bioinsecticides have storage difficulties [59], and their efficacy and persistence are strongly conditioned by the substrate and the environmental conditions of application [60]. For example, the survival of *Bb* is reduced to a few days if sprayed on synthetic surfaces as polypropylene or used in unsuitable operating conditions such as exposure to heat, UV radiation, and substrate drying, thus affecting the persistence of the insecticide product [61].

Those are among the main problems that have motivated research on precision pest management solutions, particularly lure-and-kill strategies, also for *A. albopictus* control. Among them, mechanical control methods (trapping) can be seen as a potential alternative to replace insecticides (chemical and biological) or a targeted and selective system for their delivery. Trapping would reduce the environmental impact of chemical compounds and improve control efficacy as in the objectives of precision pest management. Generally, traps have been exploited as monitoring tools, but there have been numerous attempts to transform some monitoring traps into lethal devices to be employed as mass control methods. Most monitoring traps aim to collect adult mosquitoes (e.g., gravid females or host-seeking females) or eggs by adapting their working principles to the target species, for example, by adapting odour baits and trapping method to the specific behavioural/physiological characteristics [48, 62, 63].

Traps targeting host-seeking females, such as the BG-Sentinel trap (BGS, Biogents AG, Regensburg, Germany), are usually based on semiochemical (CO_2_-based or volatile compounds such as L-lactic acid and octenol) or colour patterns that have been shown to be attractive for several mosquito species, *Aedes* in particular [64–71]. They can be equipped with active components (active traps) such as fans, sticky surfaces, or other aspiration or autocidal mechanisms to capture the lured adults [72–74], and then do not require the addition of insecticide to be converted into lethal lure-and-kill traps [75], making them potentially feasible green mass control devices. Nevertheless, despite their lower environmental impact due to the absence of pesticides, active lethal traps are not selective and are composed of non-biodegradable parts. However, among the primary limits to their use as a mass control method remain the cost of suction traps such as the BGS (ranging from tens to hundreds of dollars versus a few dollars for standard ovitraps), the need for a power supply, and the need for periodic maintenance by the user (which means further costs and reduced cost-effectiveness ratio) [76]. Then, even if upgraded to be very attractive and able to compete with a human host, these traps still have limited applicability in mass control campaigns in which the use of low-cost devices and maintenance cost reduction are crucial because a large number of traps must be deployed over a wide area to achieve a population reduction effect [77].

On the other hand, ovitraps targeting gravid females (e.g., the traditional ovitrap), one of the most widely employed and inexpensive passive surveillance/monitoring tools for detecting the presence of *A. albopictus*, already use an approach based on the characteristics of *Aedes* oviposition behaviour. They exploit the propensity of container-breeding mosquitoes to lay their eggs in small [71] artificial water collections, providing an oviposition site similar to those available in urban contexts [78]. These artificial oviposition sites have been converted from monitoring to a lethal ovitrap (LOT) [79] by adding an insecticidal ingredient (chemical or biological) [80], obtaining a low-cost device proven (under certain conditions) to reduce mosquito population density in large-scale campaigns [48]. However, their efficacy is strongly dependent on public engagement and larval source reduction from the environment [62, 81]. LOT efficacy can increase when enriched with organic infusions such as grass, hay, or oak, as well as NPK (nitrogen–phosphorous–potassium) fertilisers, to improve their attractivity [82, 83]. However, attractant efficacy is strongly conditioned by environmental conditions and cannot compensate for the competition such as attractants of the numerous water containers and natural breeding sites around the trap-treated areas [84]. This remains probably one of the most critical factors limiting ovitrap efficacy, but the development of oviposition stimulants could lead to even better control of mosquito populations using these traps [85].

Furthermore, even though cheaper than active traps, and thus economically more suitable for mass trapping, LOTs still need periodic servicing: water and insecticide refills to compensate for evaporation and prevent them from becoming new breeding sites, continuous monitoring, recovery, and disposal when not biodegradable. Even though biodegradable traps (e.g., Biotrap, Greenlid) [86] or larger standard traps (long-lasting traps) have been proposed as possible solutions, the presence of insecticide and the need to add larvicidal products to avoid adult emergence remain unsolved [87]. Analysing LOTs in terms of environmental profile, they are a precision pest management approach to tiger mosquito control. However, although more selective, they remain largely based on chemical insecticides [88], with consequently higher environmental impact and a lower safety profile compared to insecticide-free approaches such as active lethal traps, limiting their applicability in domestic contexts. Although biopesticides such as *Bti* and *Wolbachia* have been proved effective against *Aedes*, no specific improvements to traps (e.g., trap design or oviposition substrate modifications) have been proposed to promote their use or to employ other active substances [79, 81].

Therefore, although less expensive, currently available LOTs are in several cases not sufficiently cost-effective to effectively compete with chemical control methods for *A. albopictus* control or are not entirely environmentally friendly. The cost-effectiveness of ovitraps is affected by their attractiveness and the trap's active period (i.e., how much and for how long the traps are attractive) [62]. The first aspect has been improved by enhancing oviposition substrate attractiveness (e.g., by using oviposition-promoting substances), thus increasing the number of oviposited eggs [89]. More than 100 attractive substances have been found, as reported in the literature. However, they are not selective, have efficacy limited to specific environmental conditions [90, 91], and, overall, no information is available about their effect on trap competitiveness against natural breeding sites.

On the other hand, synthetic hydrogels have already been employed (together with other hydrophilic or absorbent materials, such as zeolites) to reduce servicing by limiting evaporation and trap drying. At the same time, hydrogels have been exploited for controlled-release pesticides [92, 93]. However, as currently proposed, synthetic hydrogels do not help to reduce the employment of chemical substances, for example, by effectively integrating and delivering bioinsecticides, as they are generally not biocompatible due to possible toxicity of materials and/or preparations, stressful processing conditions such as thermal or mechanical stress, or simple chemical incompatibility [94, 95]. Synthetic hydrogel substrates currently employed have not been designed to reproduce main natural breeding sites features. To the best of our knowledge, studies focusing on the effects of their chemical composition/physical features on oviposition preference and number of eggs laid are not available in the literature.

Also, the issue of dispersing a non-biodegradable and potentially dangerous synthetic polymer into the environment is not taken into account, therefore incurring the limitations of use that contradict the assumptions on which the approach we propose relies [96]. Even in the case of biodegradable compounds, their use could not entirely prevent the generation of possible contamination or toxicity due to the presence of small molecules (e.g., free radicals or reactive moieties) dispersed as degradation by-products. Consequently, very few compounds in the list of potential BPs could be considered biodegradable or not toxic, and even less could be used to produce fully biodegradable hydrogels. Moreover, nowadays, the impact on the environment in terms of CO_2_ emissions of these polymers' production process should also be considered. Hence, taking into account the problems mentioned above, for the targeted application, we suggest the use of nature-derived polymer hydrogels (starch or cellulose derivative-based) excluding sugar-based BPs (e.g., polylactic acid [PLA] or polyhydroxybutyrate [PHB]).

In particular, for *Ae. albopictus*, the biomimetic lure-and-kill approach based on BP hydrogels does not provide a new type of trap in itself, but presents a possible solution in the optimisation of currently available trapping devices. It allows both the mimicking of typical oviposition substrates and, at the same time, hosts (i.e., grown until lethal concentrations) a bioinsecticide by exploiting biocompatibility and microenvironmental features to improve efficacy, reduce servicing, and enhance environmental safety at the same time. It bypasses the use of attractants, trying to compete with natural breeding sites, identifying and imitating their key features to develop a new artificial oviposition substrate. Even better, a BM can also be tuned to create a mechanical entrapment effect, potentially making unnecessary the presence of a biocide in a trapping device [97].

## Specialisation of the biomimetic lure-and-kill approach to produce an oviposition substrate for *Aedes albopictus*

Mosquito oviposition is a complex and multifactorial event requiring selection of the egg-laying site during which the gravid female, guided by several stimuli, identifies the most suitable site for the offspring's survival [91]. Given the extreme adaptability of *A. albopictus* at the larval level [77], both in natural and in anthropised contexts of different geographical areas, many signals (e.g., surface features, volatile compounds from decaying vegetal matter and microbial community, site/background colour, adequate pH and salinity, presence of conspecific larvae or predators [40]) play a role in identifying microenvironments for site selection [98–102]. It is possible to produce an artificial oviposition substrate considering a defined number of key oviposition drivers, with constant, testable, measurable, and reproducible physical characteristics, making the substrate biomimetic, free from repellent factors, and consequently highly attractive for gravid mosquito females.

A possible technical solution for creating a substrate with the previously described properties is the use of physical or cross-linked macromolecular hydrogels based on low-cost, naturally biodegradable, and biocompatible BPs. Some suitable low-cost candidates for hydrogel composition could be cellulose, alginates, or other polysaccharides such as starch or chitosan [17]. One of the most well-known properties of macromolecular hydrogels (in particular for super-absorbent hydrogels) is the ability to absorb and retain water (or humidity from the environment for hydrophilic materials), releasing it slowly, then maintaining for an extended period the essential characteristics of a suitable *A. albopictus* breeding site. Furthermore, it is possible to regulate the gel's mechanical properties and degradation times [103], surface morphology, viscosity, pH, salinity, etc., simulating physical and chemical conditions of natural oviposition substrates of tiger mosquitoes (Table 1) and/or other container-breeding species [104].

**Table 1:** A panel of possible key parameters and substrate composition for the growth of *Beauveria bassiana* (*Bb*) and oviposition lure activity for *A. albopictus*

	pH	Salinity	Composition	Water content wt%	Substrate consistency	Surface morphology
Key parameters						
Oviposition	4–7	< 3%	Natural substrates	> 0%	Mud-like to wood	Not smooth
*Bb* growth [111]	5–7	< 3%	Natural substrates rich in sugars (e.g. cereals)	5–80 wt%	Solid substrate (fermentation)	-

**Fig. 1:**
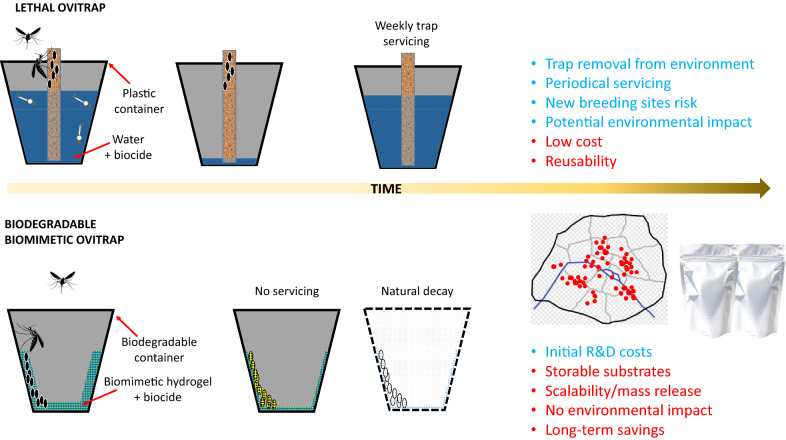
Example of a theoretical biodegradable polylactic acid (PLA) oviposition trap with biomimetic hydrogel and a bioinsecticide

In addition to the possible lure advantages due to the biomimetic design of the substrate, the use of fully biodegradable and super-absorbent hydrogel would also guarantee a technical solution to the problems of duration, maintenance, and disposal of the traps and associated costs described above. Finally, hydrogels can also be stored after lyophilisation (e.g., through freeze-drying) and subsequently rehydrated (without loss of properties), ensuring ease of use, storage, and transport [105, 106]. A so-designed hydrogel could be employed in biodegradable devices (e.g., made of PLA) or spread on cardboard-made supports (similar to glue-based sticky traps [72, 74, 107, 108]) due to the lack of liquid water in the trap. This use could ensure a possible industrial scale-up and a low-cost and easily storable final product (Fig. 1).

At the same time, these natural hydrogels can be exploited to support the survival and growth of biopesticides within the oviposition substrates by providing a suitable microenvironment for the insect pathogen growth, controlling the amount of water necessary for its survival and proliferation, avoiding desiccation, and providing nutrients that can be inserted during the macromolecular gel preparation. Entomopathogenic fungi such as *Bb* or *Metarhizium anisopliae* are interesting (but not exhaustive) examples of potentially embeddable biopesticides. The feasibility of embedding these insect pathogens has already been proven [77–79], particularly at the conidial stage. Usually, conidia are suspended in biopolymeric solutions (e.g., alginate), able to form micro shells or spheres containing the pathogens and to preserve their vitality and persistence when applied (usually by spraying after rehydration) [28, 109]. However, the polymers employed in these solutions have only a protective function and are not developed to support conidial growth. In contrast, considering the link between conidial growth and the presence of water, nutrients, and specific ranges of pH and temperature (pH 4–8; 25 °C) [110, 111], a hydrogel not only can provide protection for the conidia but could also be a substrate potentially able to promote fungal growth. By adding bioinsecticides inside a hydrogel in a trapping device, the efficiency and possibilities for use of the trapping approach can be increased, but it would also benefit from using lower starting fungal concentrations. A shortlist of some possible key physical and chemical parameters and relative ranges suitable for both lure and bioinsecticide growth (e.g., for *Bb*) are reported in Table 1. This way, it could provide a potentially effective biomimetic lure-and-kill substrate: a cellulose hydrogel with mosquito oviposition lure activity containing a living biocide (*Bb* conidia but even *Bti* spores). The only constraint is an overlapping between the proliferation condition of the biocide needed and the attractiveness of the substrate for the mosquito (Table 1).

**Fig. 2:**
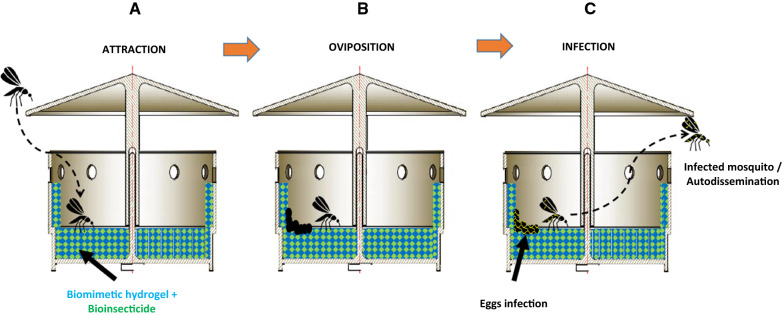
Example of a fully biodegradable auto-dissemination trap. **a** Lure phase; **b** oviposition phase; **c** lethal infection of eggs and adult and auto-dissemination phase. Finally, traps will naturally decompose due to the biodegradability of the materials: polylactic acid (PLA) and biopolymer-based biomimetic gel

Another possible advantage of including a biopesticide inside an oviposition substrate is the presence of the so-called auto-dissemination mechanism of the insecticide spread by insects after oviposition (Fig. 2). This mechanism can boost the effect of the bioinsecticide, especially for insects that do not lay all eggs in a single site (skip oviposition), such as *A. aegypti* or *A. albopictus* [112, 113].

Finally, if properly designed, hydrogel-based substrates could also perform a mechanical trapping action of eggs/larvae, exploiting their viscoelastic properties (e.g., by varying the rheological properties), eliminating the need for a biocide substance. This solution would increase the safety of the trapping device and consequently the number of scenarios in which the device could be adopted. In addition, the absence of the biocide further reduces the costs by eliminating the potential need for biocide registration. A substrate with these characteristics has already been developed, and preliminary laboratory testing shows promising results [114].

## Conclusions

The increasing problems associated with the reduced efficacy and high ecological impact of traditional pest management methods highlight the need to adopt precision methods, focusing on effective and sustainable new tools. The approach proposed herein, modulated by TE and based on the principles of biomimicry, identifies BMs as a useful tool since they are among the few (if not the only) materials capable of satisfying multiple needs at the same time (e.g. biomimetic lure, mechanical lethality, biocompatibility, and bioinsecticide growth).

As described here for the tiger mosquito, the approach has the potential for applicability to several pest species and is already technically feasible, as all the technologies necessary to design and configure materials with specific characteristics already exist.

This approach might be cost-competitive, increasing the effectiveness of substrates and devices as much as possible. This result can be obtained by identifying the most promising behavioural habits of the target species to obtain substrates able to mimic the nature and conditions necessary to obtain a specific response in the target species, exactly as is done in the scaffold–cell interaction of TE approaches.

The role of material engineering is instrumental in finding the best materials and fitting processing, technically and economically, in a continuous transfer of knowledge from materials engineering to entomology. Developing a new pest control approach requires a multidisciplinary process, and strong interaction among different research areas is needed. In fact, one of the possible limitations could be finding specific behavioural patterns in insects and quantifying their driver parameters. For example, knowledge in entomology, mycology, and materials engineering is crucial to realise the proposed tool for *A. albopictus*. Further works are necessary to verify (i) which hydrogel characteristics (e.g., humidity, pH, salinity, composition) mainly influence the oviposition behaviour and in what range of values; and (ii) the oviposition preference of *A. albopictus* for hydrogel composition compared to natural larval habitats. The final aim is to obtain the best lethality results with lower environmental impact, providing pest control tools with the highest safety and a broad application scenario [96].

## Data Availability

The data that support the findings of this study are available from the corresponding author upon request.

## Abbreviations

BM: Biomaterial
BP: Biopolymer
TE: Tissue engineering
*A. albopictus*: *Aedes albopictus*
*Bb*: *Beauveria bassiana*
LOT: Lethal ovitrap
